# Effects of Semiconductor Laser Irradiation on Differentiation of Human Dental Pulp Stem Cells in Co-Culture with Dentin

**DOI:** 10.3390/dj12030067

**Published:** 2024-03-05

**Authors:** Masafumi Yarita, Kayoko Kitajima, Takao Morita, Koichi Shinkai

**Affiliations:** 1 Advanced Operative Dentistry-Endodontics, The Nippon Dental University Graduate School of Life Dentistry at Niigata, 1-8 Hamaura-cho, Chuo-ku, Niigata 951-8580, Japan; m.yarita@ngt.ndu.ac.jp; 2 Department of Endodontics, The Nippon Dental University School of Life Dentistry at Niigata, 1-8 Hamaura-cho, Chuo-ku, Niigata 951-8580, Japan; kitajima@ngt.ndu.ac.jp; 3 Department of Biochemistry, The Nippon Dental University School of Life Dentistry at Niigata, 1-8 Hamaura-cho, Chuo-ku, Niigata 951-8580, Japan; moritat@ngt.ndu.ac.jp; 4 Department of Operative Dentistry, The Nippon Dental University School of Life Dentistry at Niigata, 1-8 Hamaura-cho, Chuo-ku, Niigata 951-8580, Japan

**Keywords:** photobiomodulation therapy, semiconductor laser, cell proliferation, cell differentiation, odontoblast-like cells

## Abstract

This study aimed to determine the effect of photobiomodulation therapy induced by semiconductor laser irradiation on human dental pulp stem cell (hDPSC) proliferation and their differentiation into odontoblast-like cells (OLCs). The effects of various semiconductor laser irradiation conditions on hDPSCs were examined. Three groups were evaluated: a single laser irradiation at 6 h post-seeding, multiple laser irradiations up to four times every 4 days after the first dose, and a control with no laser irradiation. The cells were irradiated at 10, 30, and 150 mW using a semiconductor laser. The effect of laser irradiation on hDPSC differentiation into OLCs was also determined. Four groups were evaluated, including co-culture using basic medium and dentin discs, simple culture using OLC differentiation-inducing medium, co-culture using OLC differentiation-inducing medium and dentin discs, and control culture with basic medium. The expression of the *nestin*, *ALP*, *DSPP*, and *DMP-1* genes was measured using real-time PCR. The multiple irradiation group irradiated at 30 mW exhibited significantly more cell proliferation than the control. The expression of *nestin* associated with differentiation into OLCs during each culture period tended to be lower, whereas *DSPP* and *ALP* expression was higher compared with that of the control. Multiple laser irradiations at a low power of 30 mW induced significant hDPSC proliferation and might induce differentiation into OLCs.

## 1. Introduction

Lasers are used in various areas of dentistry. Their effects on vital tissues depend on the absorption characteristics and output energy of the laser. At a higher output energy, tissue surface-absorbing lasers with high absorption to water are primarily used for the incision or ablation of soft tissues or cutting hard tissues, whereas tissue-transmitting lasers with a low absorption in water are primarily used for the incision of soft tissues and pain relief [1]. These applications in dentistry are based on the thermal or photochemical effects of lasers on living tissues [2]. The irreversible changes in tissue caused by high-powered lasers are caused by a high-level laser therapy (HLLT) effect, whereas changes also occur around the tissue where irreversible changes occur. Such changes are reversible and are caused by a low-level laser therapy (LLLT) effect [3].

LLLT, which activates tissues and cells at a power <500 mW to promote healing and reduce pain, is known as photobiomodulation therapy (PBMT) [4,5]. PBMT uses the beam of an LED light illuminator as well as a laser [6,7]. Recently, PBMT has also been used for tissue regeneration and has gained interest in the field of regenerative therapy [8,9]. PBMT may be caused by the photochemical action of lasers; however, its mechanism of action at the cellular level has not been established. A study examining the PBMT effect of a laser using a cell culture model revealed that laser irradiation enhances cell differentiation and proliferation [10]. The underlying mechanism has been shown to involve increased cell proliferation through the activation of cytochrome c in the mitochondria and MAPK/ERK signaling [11,12], although the details remain unclear.

In the field of restorative dentistry, lasers are used in cavity preparation for removing caries as well as hypersensitivity treatment. The laser cutting of rat teeth with an Er: YAG laser causes reversible pulp damage that heals with significant tertiary dentin formation [13]. Clinical studies also indicate that hypersensitivity treatment using semiconductor lasers has a suppressive effect and the irradiation of fibroblasts with semiconductor lasers results in significant cell activity [14]. In addition, laser irradiation after pulp capping increases the formation of repaired dentin [2,14]. These may be caused by the PBMT effect of the laser.

PBMT uses a low-power laser or LED light beams and represents a promising treatment for tissue regeneration because it avoids tissue and cell damage caused by heat generated during light irradiation. Human dental pulp stem cells (hDPSCs) isolated and cultured from healthy teeth exhibit pluripotency [15] and have been used in studies of the effects of PBMT on pulp cell proliferation and differentiation [16,17]. A study that examined the proliferation and differentiation of hDPSCs by direct low-power laser irradiation revealed no effect of PBMT [18]; however, in clinical practice, laser irradiation is rarely applied directly to the pulp tissue, but is often applied through dentin or some other material. Few studies have examined the effect of laser irradiation on hDPSCs when irradiated through dentin in vitro [7,18]. To our knowledge, there are no reports describing an effect of laser irradiation on hDPSCs during co-culture with dentin pieces. It is possible to determine the ability of PBMT to promote hDPSC differentiation into odontoblast-like cells by adding bioactive substances for collagen fiber organization and differentiation to the culture medium during co-culture with dentin fragments.

In this study, we performed laser irradiation of hDPSCs cultured under different conditions using a semiconductor laser and determined the effects on the proliferation and differentiation of the hDPSCs into OLCs in vitro. The aim of this study was to determine the effect of PBMT induced by semiconductor laser irradiation on the activity of hDPSCs and their differentiation into OLCs. The null hypothesis is “semiconductor laser irradiation would not affect the cellular activity of hDPSCs and the promotion of their differentiation into OLCs”.

## 2. Materials and Methods

### 2.1. Cell Culture

hDPSCs (Lonza Group Ltd., Basel, Switzerland) between 4 and 6 passages were used for the experiments. Dulbecco’s modified Eagle’s medium (DMEM, Sigma-Aldrich, St Louis, MO, USA) containing 10% fetal bovine serum (FBS, Cytiva, MA, USA) plus antibiotics (penicillin, streptomycin, and amphotericin B) was used as the basic medium (BM). The cell culture was performed at 37 °C in 5% CO_2_ under humidified environment. The medium was replaced every 3 days.

**Figure 1:**
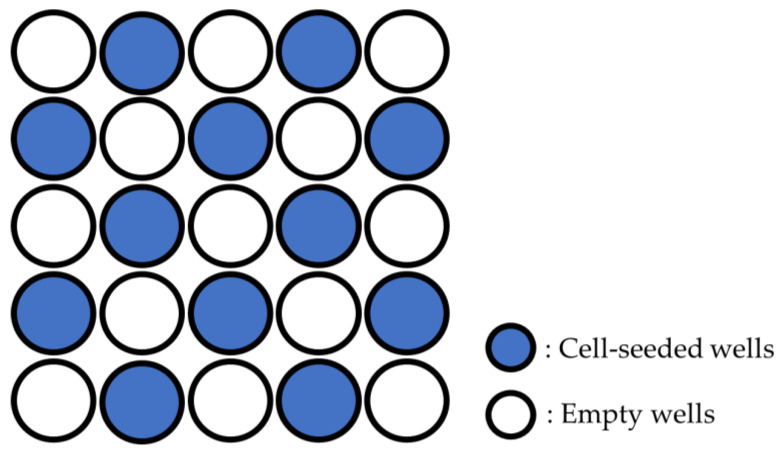
Location of wells seeded with cells.

hDPSCs were seeded in BM at a concentration of 1.0 × 10⁴/mL per well of a 96-well plate for the cell proliferation assay, and at a concentration of 5.0 × 10⁴/mL per well of a 48-well plate for quantitative reverse transcription PCR (qRT-PCR). The cells were seeded in wells at positions skipping every other well, front to back and left to right, so that the cells seeded in adjacent wells would not be irradiated by the laser (Figure 1).

### 2.2. Laser Irradiation

**Figure 2:**
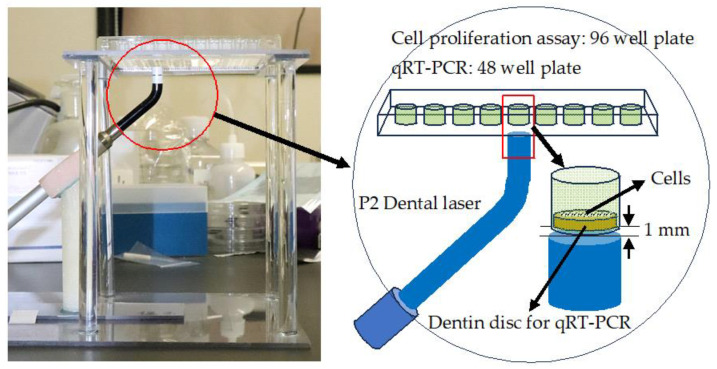
Homemade laser irradiation system.

A semiconductor laser (P2 Dental Laser System, Pioon Laser Technology Co., Wuhan, China) was used to irradiate the plates at a continuous wavelength of 650 nm for 40 s. This laser apparatus can irradiate laser beams of 650 nm and 810 nm. In the present study, we used a wavelength of 650 nm because a wavelength of 630–670 nm is suitable for PBMT. Laser irradiation was performed using the homemade device shown in Figure 2. Using the homemade stand and arm, the laser handpiece and well plate were fixed with the irradiated surface of the laser tip 1 mm away from the bottom of the well and facing upward. The diameter of the irradiated spot was 8 mm.

For the cell proliferation assay, a 96-well plate was used and cells were seeded directly on the bottom of the wells. For qRT-PCR, a 48-well plate was used, and cells were seeded on dentin discs placed at the bottom of the wells.

### 2.3. Cell Proliferation Assay

The first irradiation was performed 6 h after seeding, and two experimental groups were established: the multiple laser irradiation group (LM) with up to four radiation exposures every 4 days, the single laser irradiation group (LS) only exposed to the first irradiation, and the group without laser irradiation (WL) for each experimental group. The laser power was set at 10, 30, and 150 mW, and the output was adapted to each experimental group (*n* = 7) (Table 1).

The number of cells in each experimental group was counted on days 1, 3, 5, 7, 10, and 14 after seeding. The alamarBlue^®^ Cell Viability Reagent (Invitrogen Corp., Carlsbad, CA, USA) was added to the wells of each group and incubated for 2 h at 37 °C. Fluorescence (595 nm) was measured using a plate reader (Spectra Max, Molecular Devices, CA, USA), and the cell count was calculated from the fluorescence intensity.

### 2.4. Preparation of Dentin Discs

Dentin discs were prepared using bovine mandibular anterior root dentin. First, the crown and root portions were cut using a precision cutting machine (Isomet, Buehler, Lake Bluff, IL, USA), and the root portions were cut perpendicular to the tooth axis with a thickness of approximately 6 mm to produce a ring-shaped root fragment. The root fragments were cut longitudinally and adjusted to a dentin plate with a thickness of approximately 200 µm. A 5 mm diameter dentin disc was prepared from the dentin plate using a trephine bar. The prepared dentin discs were washed with distilled water, immersed in 17% EDTA for 1 min to remove the smear layer on the dentin discs, and washed again with distilled water. The dentin discs after EDTA treatment were sterilized using an autoclave (2 atm, 121 °C, 15 min).

### 2.5. Differentiation of hDPSCs into OLCs

Ascorbic acid (50 μg/mL), dexamethasone (0.1 μM), β-glycerophosphate (5 mM), and TGF-β (10 ng/mL) were added to the basic medium to establish an OLC differentiation-inducing medium (ODM). The medium was changed every three days and the cultures were incubated at 37 °C in a humidified environment containing 5% CO_2_.

Four experimental groups were established: a control in which simple culture was performed using BM (C); a group in which co-culture was performed using BM and dentin discs (D); a group in which simple culture was performed using ODM (O); and a group in which ODM and dentin discs were applied (OD) (Table 2). All experimental groups were exposed to laser irradiation with an output of 30 mW. The first irradiation was performed 6 h after seeding, with up to six radiation exposures every 4 days (*n* = 8).

### 2.6. qRT-PCR

The gene expression of nestin, alkaline phosphatase (ALP), dentin sialophosphoprotein (DSPP), and dentin matrix protein 1 (DMP-1) was measured after 1, 2, and 3 weeks using qRT-PCR (Table 3). Total RNA was isolated using a total RNA purification kit (NucleoSpin^®^ RNA, Takara Bio Inc., Shiga, Japan) according to the manufacturer’s protocol and quantitated using a NanoDrop™ 2000c spectrophotometer (Thermo Fisher Scientific, Waltham, MA, USA). Quantitative PCR assays were performed using the One Step TB Green^®^ PrimeScript™ RT-PCR Kit II (Takara Bio Inc.) and the StepOnePlus Real-Time PCR System (Applied Biosystems, Thermo Fisher Scientific, Waltham, MA, USA). ΔΔCt values were used to determine relative gene expression levels.

### 2.7. Statistical Analysis

The data were analyzed by a one-way ANOVA and Tukey’s post hoc test or a Kruskal–Wallis test with a Steel–Dwass post-hoc test at a 5% significance level according to equal variances to determine significant differences among the experimental groups. BellCurve for Excel (Version 3.21, Social Survey Research Information, Tokyo, Japan) was used for statistical analysis.

## 3. Results

### 3.1. Cell Proliferation of hDPSCs with LS or LM

**Figure 3:**
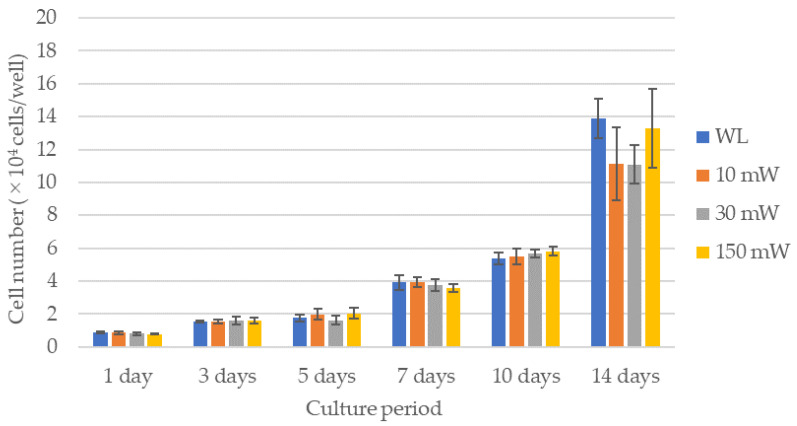
Results of the cell proliferation assay in single laser irradiation group (LS). There were no significant differences between the experimental groups over all the culture periods. WL: group without laser irradiation, 10 mW: group irradiated with 10 mW laser, 30 mW: group irradiated with 30 mW laser, 150 mW: group irradiated with 150 mW laser.

**Figure 4:**
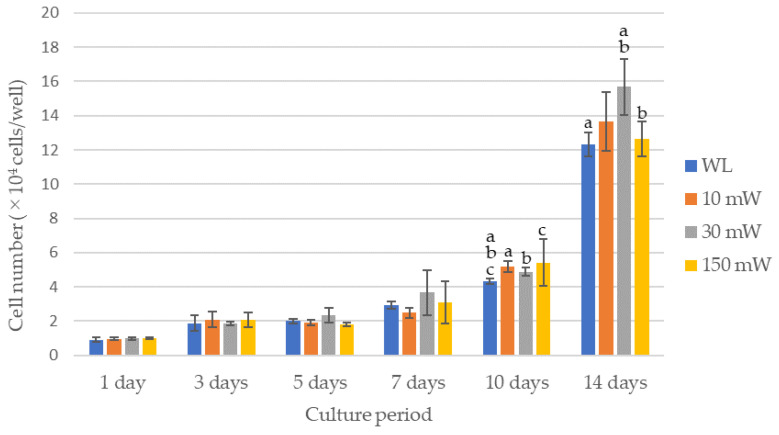
Results of the cell proliferation assay in multiple laser irradiations group (LM). The same lowercase letters indicate a significant difference between the experimental groups during each culture period (*p* < 0.05) (a–c). WL: group without laser irradiation, 10 mW: group irradiated with 10 mW laser, 30 mW: group irradiated with 30 mW laser, 150 mW: group irradiated with 150 mW laser.

The results of the cell proliferation assay are shown in Figure 3 and Figure 4. The LS (single laser irradiation) caused an increase in the cell counts over time for all the experimental groups; however, there were no significant differences between the experimental groups over all the culture periods. No significant differences in the cell counts among the experimental groups were observed by LM (multiple laser irradiations) until 7 days after culturing, whereas after 10 days, all the experimental groups exhibited significantly higher cell counts compared with the control (*p* < 0.021). A comparison of the cell counts after 14 days of culture revealed that the cell count of the 30 mW irradiation group was significantly higher compared with that of the control or the 150 mW irradiation group (*p* < 0.021). The 10 mW and 150 mW irradiation groups also had a higher cell count than the control, but there were no significant differences between them.

### 3.2. Gene Expression of hDPSCs Co-Cultured with Dentin Disc in BM or ODM

**Figure 5:**
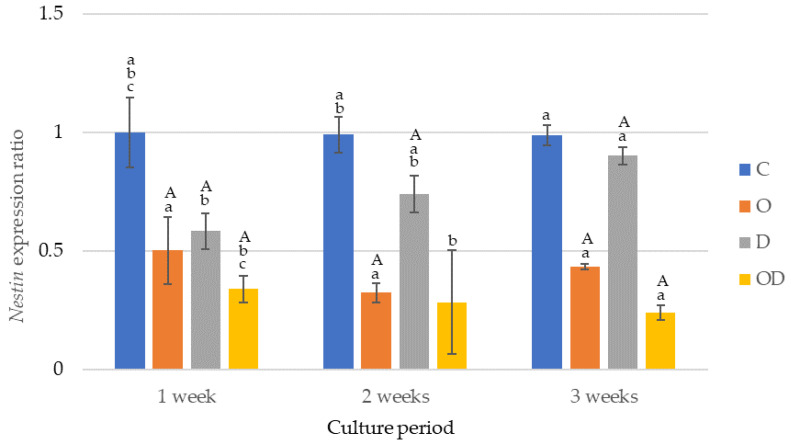
Results of *nestin* gene expression. The same lowercase letters indicate a significant difference between the experimental groups during each culture period (*p* < 0.05) (a–c). The same uppercase letter indicates a significant difference between the different culture periods for each experimental group (*p* < 0.05) (A).

The results of the *nestin* gene expression are shown in Figure 5. The *nestin* gene was expressed the highest in the control during all the culture periods after cell seeding, with significant differences in the experimental groups except between O and D after 1 week of culture, and O and OD after 2 weeks of culture (*p* < 0.009). O exhibited a significant decrease in gene expression after 2 and 3 weeks of culture (*p* < 0.003), whereas a significant increase occurred between 2 and 3 weeks of culture (*p* = 0.002). OD exhibited a significant decrease in gene expression after 3 weeks of culture (*p* = 0.017). Conversely, D showed a significant increase in the expression after 2 and 3 weeks of culture (*p* = 0.017).

**Figure 6:**
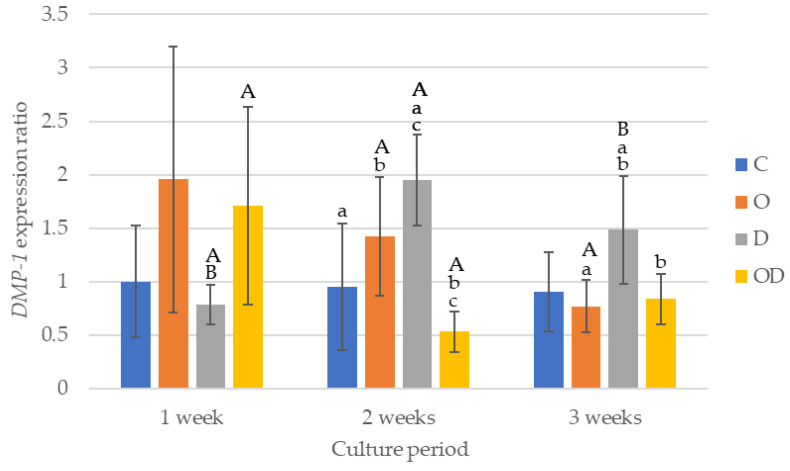
Results of *DMP-1* gene expression. The same lowercase letters indicate a significant difference between the experimental groups during each culture period (*p* < 0.05) (a–c). The same uppercase letters indicate a significant difference between the different culture periods for each experimental group (*p* < 0.05) (A,B).

The results of DMP-1 gene expression are shown in Figure 6. After 1 week of culture, there were no significant differences among all the groups. After 2 weeks of culture, the gene expression was higher in the following order D > O > C > OD. Comparing the gene expression among the experimental groups after 2 weeks of culture, D was significantly higher than C and OD (*p* < 0.043), and O was significantly higher than OD (*p* = 0.017). After 3 weeks of culture, D was the highest, with a significant difference between D and O, and D and OD (*p* < 0.024), whereas no significant difference was observed among C, O, and OD. D exhibited a significantly higher gene expression after 2 and 3 weeks of culture compared with 1 week of culture (*p* < 0.009), whereas the gene expression after 3 weeks of culture was slightly lower than that after 2 weeks of culture. The gene expression of O showed a gradually decrease with a significantly lower gene expression after 3 weeks of culture compared with 2 weeks of culture (*p* = 0.002). For OD, the gene expression after 1 week of culture was the highest with a significant difference compared with 2 weeks of culture (*p* = 0.038).

**Figure 7:**
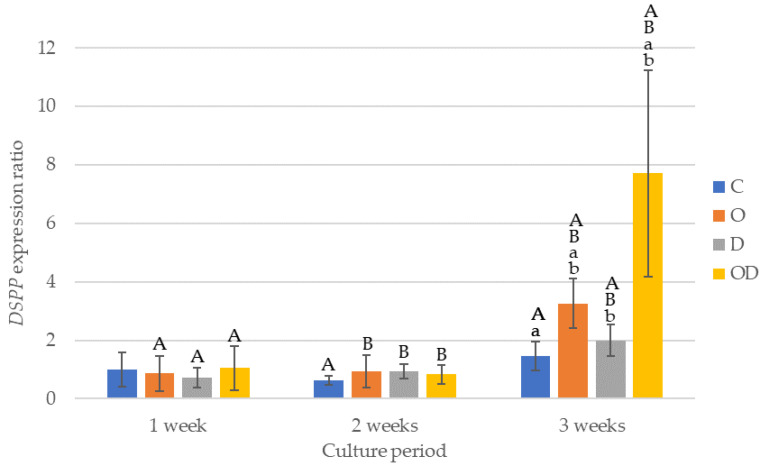
Results of *DSPP* gene expression. The same lowercase letters indicate a significant difference between the experimental groups during each culture period (*p* < 0.05) (a,b). The same uppercase letters indicate a significant difference between the different culture periods for each experimental group (*p* < 0.05) (A,B).

The results of *DSPP* gene expression are shown in Figure 7. There was no significant difference among the experimental groups after 1 and 2 weeks of culture. After 3 weeks of culture, OD showed the highest gene expression with a significant difference compared with the other groups (*p* < 0.012), and O showed higher gene expression compared with C and D (*p* < 0.017). All the experimental groups exhibited a trend of low gene expression ratios until after 2 weeks of culture, whereas the gene expression ratio after 3 weeks of culture was significantly higher compared with that after 2 weeks of culture (*p* < 0.001), particularly in O and OD. In the control, a significant difference was detected between 2 and 3 weeks of culture.

**Figure 8:**
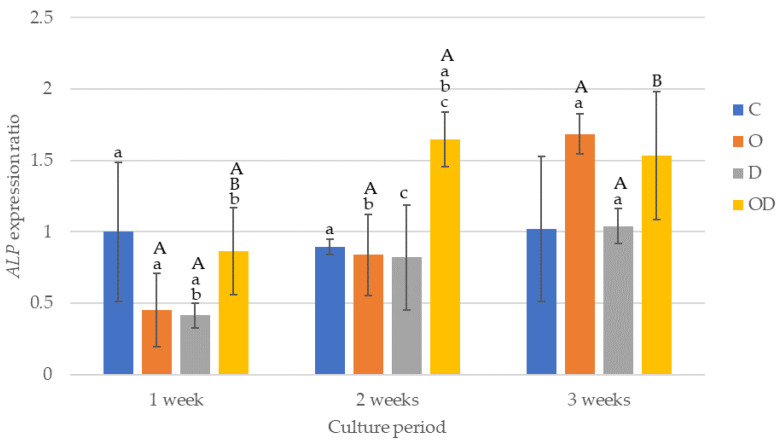
Results of *ALP* gene expression. The same lowercase letters indicate a significant difference between the experimental groups during each culture period (*p* < 0.05) (a–c). The same uppercase letters indicate a significant difference be-tween the different culture periods for each experimental group (*p* < 0.05) (A,B).

The results of ALP gene expression are shown in Figure 8. After 1 week of culture, the gene expression was higher in C and OD, and both were significantly higher compared with D (*p* < 0.017). After 2 weeks of culture, OD was significantly higher than the other experimental groups (*p* < 0.006), whereas there were no significant differences among the other experimental groups. After 3 weeks of culture, O exhibited the highest gene expression in the group, with a significant difference compared with D (*p* = 0.004), whereas no significant differences were observed when comparing the groups except for O. When comparing the culture periods for the gene expression ratios, significant differences were evident between 1 week vs. 2 weeks, and 2 weeks vs. 3 weeks (*p* < 0.001) for O, 1 week vs. 3 weeks (*p* = 0.002) for D, and 1 week vs. 2 weeks, and 1 week vs. 3 weeks (*p* < 0.003) for OD.

## 4. Discussion

The results of this study indicated that low-power laser irradiation at 30 mW significantly increased the proliferation of hDPSCs and the expression of genes associated with their differentiation into OLCs, which was different from the control during each culture period. Therefore, the null hypothesis, “semiconductor laser irradiation will not affect the cellular activity of hDPSCs and their promotion of differentiation into odontoblast-like cells” cannot be fully rejected.

Compared with the control, the cell proliferation of laser-irradiated hDPSCs showed no significant change throughout the entire culture period with single irradiation, whereas multiple irradiation treatments resulted in significantly higher cell proliferation after 10 and 14 days in culture. Thus, cell proliferation was more positively affected by multiple irradiation exposures compared with that caused by single laser irradiation at equivalent energy densities. This is likely the result of a higher total energy administered to the cells by multiple treatments. The irradiation energy density required to activate cell proliferation was reported to be 0.5–10 J/cm^2^, and cells are activated even more at an energy density of 0.5–4 J/cm^2^ [19]. In the present study, irradiation at 10 mW and 30 mW (0.8 J/cm^2^ and 2.4 J/cm^2^) for 40 s resulted in a range of 0.5–4 J/cm^2^. Laser irradiation did not lead to cell activation in the single irradiation group, but the multiple irradiation group exhibited an overall tendency for cell proliferation, with the highest levels observed at a low power of 10–30 mW, particularly at 30 mW. A previous study reported that the activation of hDPSCs occurred when irradiation was administered through multiple exposures at a lower power [20]. This is consistent with the results of the present study. Therefore, multiple irradiation treatments at a low power effectively activate hDPSCs. An output of 30 mW was considered appropriate for this experiment; thus, the laser output was set to 30 mW to evaluate the differentiation of hDPSCs into odontoblast-like cells.

To determine whether laser irradiation affects the differentiation of hDPSCs into OLCs under conditions that mimic living teeth, we established four experimental groups: culture with basic medium only (control: C), dentin co-culture with basic medium (D), culture with differentiation-inducing medium only (O), and dentin co-culture with differentiation-inducing medium (OD). All the experimental groups were irradiated. The effects of PBMT on C, dentin-mediated induction in D, OLC induction factor in O, and dentin-mediated induction factor in OD on differentiation into OLCs were analyzed.

We also examined the expression of the odontoblast markers *nestin*, *DSPP*, *DMP-1*, and *ALP* to determine whether PBMT induced by low-power laser irradiation promoted the differentiation of hDPSCs into OLCs. Because hDPSCs differentiate into a variety of cells, reliable determination requires alizarin red and immunohistochemical staining in addition to examining the expression of these genes [21]. However, under co-culture with dentin pieces, even if cells walled in with dentin were stained, the background dentin fragments would also be stained, thus preventing the cells from being identified. In addition, ultrasonic vibration procedures are necessary to separate cells from the dentin wall, but ultrasound kills the cells. Therefore, the differentiation of hDPSCs into OLCs was determined only by marker expression, which was a limitation of this study.

*DSPP* gene expression was minimal after 1 week of culture; however, significantly higher *DSPP* gene expression was observed in the O and OD groups after 3 weeks of culture compared with the control. *DSPP* is synthesized by odontoblasts and was most abundantly expressed in the experimental group co-cultured with dentin fragments in medium supplemented with an OLC differentiation-inducing factor, which suggests that laser irradiation through dentin promotes the differentiation of hDPSCs into OLCs. When hDPSCs were cultured on a scaffold of porous material, such as dentin, which has numerous dentin tubules, differentiation into OLCs occurred [8]. In the present study, the effect of dentin co-culture in promoting the differentiation of hDPSCs into OLCs was also observed, and laser irradiation promoted the differentiation of hDPSCs into OLCs.

*DMP-1*, another genetic marker of odontoblasts, is expressed during the early stages of dentin formation. hDPSCs cultured with endothelial cells and their secreted product, endothelin-1, exhibited the highest expression ratio of *DMP-1* after 1 week of culture, followed by a significant decrease after 3 weeks of culture compared with 1 week of culture [22]. In the present study, *DMP-1* expression was highest after 2 weeks of culture in O and D and decreased after 3 weeks of culture, whereas OD decreased after 2 weeks of culture and increased after 3 weeks of culture. These results are consistent with a previous report indicating a decrease after 3 weeks of culture. Interestingly, after 1 week of culture, there were 2–4 specimens in which marker expression was not detected in O and OD with differentiation-inducing medium, whereas some samples exhibited high expression rates. The reason for the large differences among specimens is unclear. Because no such differences between specimens were observed in the experimental group using the basic medium, one reason may be that the proliferation of hDPSCs was significantly suppressed in some specimens during differentiation to OLCs.

*ALP* is a membrane protein localized to plasma cells and promotes the calcification of hard tissues, such as bones and teeth. *ALP* is also increased in the early stages of this process, similar to *DMP-1* [8,23]. The results of this study, however, indicated that the expression ratio of the *ALP* marker gene was higher after 2 weeks or 3 weeks of culture compared with 1 week of culture in all experimental groups. Because *ALP* is also an osteoblast marker, it may not be explicitly recognized as a marker of OLCs, although PBMT may act to suppress *ALP* activity in hDPSCs at the early stages of differentiation.

Previous studies have reported that nestin is a gene related to odontoblast differentiation and dentin formation [24] and that it is increased in the early stages of differentiation to OLCs [25]; hence, it was used as a marker gene for odontoblasts in this study. However, the expression of nestin genes after 14 days of culture was significantly higher in C and D compared to O and OD. Because it has been reported that TGF-β, which is an OLC differentiation inducer, suppresses nestin expression [26], it is speculated that the suppression of nestin expression in O and OD compared with C and D was due to TGF-β added to the OLC differentiation induction medium. However, the causative factor for the suppression of nestin gene expression in O and OD is undefined.

A previous study was performed in which the effects of laser irradiation on a porcine dental pulp-derived cell line (PPU-7) on pulp cells and tissues using an Er:YAG laser or semiconductor laser. Laser irradiation activated matrix metalloproteinases in the pulp tissue and promoted gene expression in odontoblasts through *TGF-β1* activity [27]. Yucheng et al. also reported that rat dental pulp cultured on a Millipore transfilter scaffold containing *TGF-β1* differentiated into odontoblasts with dentin formation [28]. The results of this study indicate that hDPSCs cultured in differentiation-inducing medium containing *TGF-β1* significantly increased the expression ratio of *DSPP* markers associated with odontoblasts following laser irradiation after 3 weeks of culture compared with those cultured in basic medium without *TGF-β1*. These results suggest that low-power irradiation with a semiconductor laser promotes the differentiation of hDPSCs into OLCs because of the PBMT effect.

In daily dental practice, dentists often encounter cases of hypersensitivity. Hypersensitivity is generally treated by applying desensitizers or adhesive dental materials to the exposed dentin in the cervical region, and laser therapy has also been applied [29,30]. Tissue-penetrating semiconductor lasers and Er:YAG lasers absorbed by the tissue surface are mainly used for laser treatment of hypersensitivity [30]. The mechanism of each laser’s action on hypersensitivity differs, and the details of the mechanism remain unclear. The hypersensitivity suppression of laser therapy using semiconductor lasers may be attributed to the effect of PBMT [18,31,32]. The effect of PBMT by laser irradiation may depend on the amount of energy density received by the irradiated tissue. From the basic data of this study, it can be noted clinically that the appropriate method of laser irradiation is important for the activity of dental pulp cells.

In summary, the results of this study indicate that PBMT induced by semiconductor laser irradiation is effective at activating the proliferation of hDPSCs and promoting their differentiation into OLCs. However, it was difficult to accurately determine the differentiation-promoting effect of PBMT on OLCs in short-term cultures because hDPSCs may be in the process of differentiating into hard tissue-forming cells, such as OLCs, in short-term cultures. Long-term incubation may be needed to define this point more precisely. To confirm the differentiation of hDPSCs into OLCs, alizarin and immunohistochemical staining methods in dentin co-culture experiments should be conducted. Hence, in addition to using real-time PCR, alizarin staining and immunochemical staining were attempted; however, the cells could not be identified because they were located on the dentin fragments. Ultrasonic vibrations must be applied to the dentin fragments to detach the cells from the dentin fragments, but this was abandoned because the ultrasonic vibrations prevented the cells from surviving. This is an issue for the future. We are also considering the utilization of Western blotting and flow cytometry analysis as our next research project.

## 5. Conclusions

Multiple 30 mW low-power laser irradiations using a semiconductor laser resulted in the significant proliferation of human dental pulp stem cells and induced differentiation into odontoblast-like cells. Within the limitations of this study, the effect of low-power laser irradiation on human dental pulp stem cells may occur by photobiomodulation therapy.

## Figures and Tables

**Table 1:** Experimental groups for hDPSC proliferation test.

	LS				LM			
	WL	10 mW	30 mW	150 mW	WL	10 mW	30 mW	150 mW
6 h after seeding	−	+	+	+	−	+	+	+
4 day after seeding	−	−	−	−	−	+	+	+
8 day after seeding	−	−	−	−	−	+	+	+
12 day after seeding	−	−	−	−	−	+	+	+

LS: single laser irradiation group, LM: multiple laser irradiation group, WL: group without laser irradiation, +: laser irradiation, −: not laser irradiation.

**Table 2:** Experimental groups for differentiation of hDPSCs into OLCs.

Group	Laser Irradiation	BM	ODM	Dentin Discs
D	+	+	−	+
O	+	−	+	−
OD	+	−	+	+
C	+	+	−	−

BM: basic medium, ODM: OLC differentiation-inducing medium, D: group in which co-culture was performed using BM and dentin discs, O: group in which simple culture was performed using ODM, OD: group in which ODM and dentin discs were applied, C: control in which simple culture was performed using BM, +: Corresponding items performed. −: Corresponding items not performed.

**Table 3:** Nucleotide sequence and amplicon size.

Gene	Nucleotide Sequence	Amplicon Size (bp)
DSPP	F: GGGCAAAGGCAATGTCAAGA	160
	R: TCCTTGCATGGACTTATCATCAA	
DMP-1	F: CAAGACAGAGAGCTATGAACACGATAT	115
	R: TGCAACCTTCCAACTCCAATG	
Nestin	F: CAGGGGCAGACATCATTGGT	77
	R: CACTCCCCCATTCACATGCT	
ALP	F: CCGTCTGTGACCCATCTCATG	110
	R: AGGGCAGCCTCTGTCATCTC	
GAPDH	F: GACAGTCAGCCGCATCTTCT	104
	R: GCGCCCAATACGACCAAATC	

DSPP: dentin sialophosphoprotein, DMP-1: Dentin Matrix 1, ALP: alkaline phosphatase, GAPDH: Glyceraldehyde 3-phosphate dehydrogenase, F: forward, R: reverse.

## Data Availability

The data presented in this study are available on request from the corresponding author.
